# Family Social Capital: Links to Weight-Related and Parenting Behaviors of Mothers with Young Children

**DOI:** 10.3390/nu13051428

**Published:** 2021-04-23

**Authors:** Virginia Quick, Colleen Delaney, Kaitlyn Eck, Carol Byrd-Bredbenner

**Affiliations:** Nutritional Sciences Department, Rutgers University, New Brunswick, NJ 08901, USA; vquick@njaes.rutgers.edu (V.Q.); cld142@sebs.rutgers.edu (C.D.); kaitlyneck@gmail.com (K.E.)

**Keywords:** mothers, social capital, nutrition, behavior, child health and wellbeing

## Abstract

Family social capital includes the social relationships, values, and norms shared by a family and is positively linked with children’s mental and physical health status. This cross-sectional study addresses a gap in the literature related to family social capital vis-à-vis weight-related behaviors and home environments of 557 mothers and their young children (ages 2 to 9 years). Mothers completed an online survey comprised of valid, reliable questionnaires assessing family relationships and weight-related behavioral and home environment measures. The measures that determined family social capital (i.e., supportive, engaged parenting behaviors; family cohesion; family conflict; and family meal frequency) yielded distinct tertile groups that differed significantly (*p* < 0.001) on every family social capital measure with large effect sizes. Analysis of variance with Tukey post-hoc test revealed greater family social capital was linked to significantly better maternal health, dietary intake, physical activity, and sleep behavior. Additionally, maternal modeling of healthy eating and physical activity, child feeding practices, and home environments was higher in groups with greater family social capital. Child mental and physical health, physical activity, and sleep quality were better in families with greater family social capital. Findings suggest greater family social capital is linked to healthier weight-related behaviors and home environments. Future intervention studies should incorporate strategies to build family social capital and compare longitudinal outcomes to traditional interventions to determine the relative value of family social capital on health behaviors.

## 1. Introduction

The obesity epidemic is a serious worldwide concern that impairs the health and wellbeing of millions of adults and children. Obesity prevention is crucial, especially for children because children obesity is positively correlated with obesity in adulthood and an increased risk for comorbidities [1]. Parents play a vital role in childhood obesity prevention in that they have great influence on children’s weight-related behaviors, such as diet, physical activity, and sleep [2,3,4]. Parental influence may take many forms, such as modeling behaviors, establishing household routines, and creating home environments that support or dissuade behaviors. Family social capital is another type of influence that affects children [5,6].

Capital is typically thought of as economic (e.g., money, wealth), material (e.g., tools, possessions), and human (e.g., education, skills). Another type of capital, known as social capital, was conceptualized in Coleman’s social capital theory as the interpersonal relationships within social groups, such as families, neighborhoods, and communities, that facilitate access to resources (e.g., information, advice) needed to accrue resources and achieve certain goals [7]. The quality and amount of social capital available is positively correlated with the possibility of reaping benefits and reaching goals [8]. The effect of social capital is considered particularly crucial to providing children with access to opportunities that support their optimal development and positive outcomes [5,6,8,9,10]. Children’s social capital is transmitted primarily by the family [7].

Family social capital is built in the home and considered one of the most powerful, enduring types of social capital [11]. It includes the social relationships, values, and norms shared by a family and is created through emotionally warm home environments that promote parent:child engagement, family cohesion, and lasting affectionate attachments [6,12]. Family social capital is also developed by teaching children behavioral norms that facilitate their successful integration into other social structures, such as schools, workplaces, and the community [12]. For children, their social capital is clearly associated with the degree to which they can access, trust, and benefit from parents’ human capital (i.e., parent education, skills, values, social competence). Factors that can compromise family social capital include physical, emotional, and/or mental disengagement between parents and children (e.g., parental absence from the home, extensive parental employment commitments, parents’ preference for adult pursuits, parent illness, children’s extensive use of media or engagement with social groups outside the family), household conflict, and/or lack of clearly communicated and reinforced rules about acceptable, prosocial norms and behaviors [13,14].

Family social capital influences the health and well-being of children [5,6]. For example, a comprehensive systematic review and meta-analysis found 55 studies investigating relationships between family social capital and children’s mental health and problem behaviors [5]. The researchers concluded that family social capital was positively linked with children’s self-esteem and negatively associated with anxiety, depression, suicide ideation, and engaging in anti-social behaviors such as aggression and defiant actions [5]. The 21 studies investigating general health status that were analyzed in this same systematic review revealed that children and adolescents in families with higher levels of social capital had better overall health status, higher quality of life, and greater wellbeing [5].

Despite the obesity epidemic and the intense research effort to identify factors that protect against excess weight gain, little attention has focused on associations between family social capital and weight-related behaviors, such as dietary intake and physical activity [5,15]. In fact, McPherson et al.’s systematic review of family social capital’s influence on children located just two studies addressing nutrition, three studies examining physical activity, and two studies investigating weight status [5]. The systematic review conducted by Alvarez et al. identified a single study related to nutrition behaviors in children and family social capital [6]. The scant evidence available suggests that higher levels of family social capital are associated with better nutritional outcomes, greater physical activity, and better weight status [5,6].

It is surprising that so few studies have considered how family social capital is associated with weight-related behaviors. Equally surprising is the lack of attention given to family social capital and parental weight-related behaviors given parents are children’s role models, are family food gatekeepers, and create the structure/lifestyle environment within the home; thereby, strongly influencing weight-related behaviors of children that track into adulthood [2,3,4,16,17,18,19,20,21,22]. Thus, this exploratory, secondary analysis reported here was conducted in response to the dearth of research related to family social capital vis-à-vis weight-related behaviors and home environments of parents and children. Based on prior work [5,6,15], it was hypothesized that family social capital would be significantly associated with positive weight-related behaviors and healthy home environments of mothers with young children. A sound understanding of the associations between family social capital and health, weight-related behaviors, and home environments has the potential to inform the development of more effective health, nutrition, and obesity prevention interventions.

## 2. Materials and Methods

The Institutional Review Board at the authors’ university approved this investigation (Protocol #11-294). This secondary analysis used data collected at baseline (pre-randomization) for the HomeStyles randomized controlled trial [23,24].

### 2.1. Sample

Participants were recruited with electronic announcements, flyers, and in-person invitations to participate in a program to “build even happier, healthier, safer families”. Recruitment announcements were distributed in locations frequented by parents, such workplace listservs, community settings (e.g., farm markets), and school-related activities. A study recruitment company also assisted with participant recruitment. All participants gave informed consent and were compensated $15 after completing the study baseline survey.

Eligibility criteria for this secondary analysis were aged 20–45 years, parent of one or more children between the ages of 2 to <9 years, ability to read English, made all or most decisions about family food choices, lived in study defined New Jersey or Arizona (study catchment area), had regular access to the Internet, and gave credible answers (e.g., did not answer all questions in a series similarly). Fathers were not included in this analysis a due to too few responses. Of the 5494 individuals who visited the study survey website, 557 met all eligibility criteria, gave informed consent, and completed the entire survey.

### 2.2. Instruments

Data were collected using the “**H**ome **O**besogenicity **M**easure of **E**nvironment**S**” (HOMES) online survey. This survey was comprised of valid, reliable questionnaires described in detail elsewhere [23,24,25]. To summarize, participants reported sociodemographic characteristics (e.g., age, race/ethnicity, highest education completed children under age 18 years in the household, maternal employment, family affluence). Participants also completed scales assessing maternal and child health and weight-related behaviors, parenting behaviors, and family social capital.

Maternal and child health status was evaluated using the Health Quality of Life assessments for health status, physical health, and mental health from the Centers for Disease Control and Prevention [26,27]. The Patient Health Questionnaire (PHQ-2) assessed maternal depression severity [28]. Maternal stress level was assessed with Cohen’s brief Perceived Stress scale (PSS-2) [29].

Maternal and child weight-related behaviors included dietary intake, physical activity, and sleep. Dietary intake behaviors focused on an indicator of healthy behaviors (i.e., fruit/vegetable servings/day) and an indicator of unhealthy choices (i.e., sugar-sweetened beverage servings/day). Block’s Fruit/Vegetable Screener assessed daily fruit/vegetable intake for mothers and daily fruit/vegetable juice intake for children [30]. Daily servings of sugar-sweetened beverages (i.e., soft, fruit, tea, coffee, and energy drinks) were measured using the HOMES Drinks Intake Screener [25]. The HOMES Physical Activity Questionnaire assessed physical activity level with sedentary behavior evaluated using an indicator item of total sedentary screen time duration daily [25,31]. Pittsburgh Sleep Quality Index components evaluated overall sleep quality and total duration of sleep each night [32,33].

Parenting behaviors focused on modeling behaviors, child feeding practices, and home environment conditions [23,24,25]. Mothers reported their modeling of healthy eating and physical activity behaviors to children. Child feeding practices evaluated were restriction of children’s access to low nutrient density foods, pressure on children to eat healthy foods, and instrumental feeding (i.e., offering children palatable food as a reward for eating healthy foods). Home physical environment conditions included physical activity and food availability. The HOP-Up questionnaire was used to evaluate physical activity space and supports inside homes and in the outdoor areas immediately outside homes [34]. Household availability of 100% fruits/vegetable juice and sugar-sweetened beverages were assessed with the Household Food Supplies Questionnaire [35].

Family social capital is evaluated in various ways, with no standard method for determining it [6,36,37]. The measures used in this study were modeled on previous research [6,12,36,38,39,40,41,42,43] and included family social environment indicator scales assessing supportive, engaged parenting behaviors; family cohesion; family conflict; and frequency of family interactions. The mother:child verbal engagement scale contained 2 items that assessed the frequency mothers talked with children. The single item mother:child physical engagement scale evaluated physical warmth (i.e., I give my kids lots of hugs and kisses). Both engagement scales were modeled on the Earls et al.’s home and life interview survey [44]. Family cohesion and family conflict constructs from the brief Family Environment scale assessed feelings of family support and togetherness (e.g., there is a feeling of togetherness in our family) and family disapproval and disputes (e.g., family members often criticize each other) with 2 and 3 items, respectively [45,46,47]. The Family Meal Frequency scale was analogous to measures used by De Clercq et al. [39]. This scale assessed how many times each week families interacted by eating meals together (breakfast, lunch, and dinner). Family meals are considered a “fundamental aspect of family life that offers the opportunity of socialization” [39], with frequency of these meals considered a gauge of family closeness [48]. All family social capital scales, except Family Meal Frequency, had these 5 answer choices: Strongly disagree, disagree, neither agree nor disagree, agree, strongly agree, scored 0 to 4, respectively, with scoring reversed for negatively worded statements. The Family Meal Frequency questionnaire included all meals (breakfast, lunch, and dinner) eaten as a family each week with answers scored as 0 to 4, respectively, if families ate together <5 meals/week, ≥5 to <9 meals/week, ≥9 to <13, ≥13 to <17 meals/week, or ≥17 meals/week. For all family social capital scales, a higher scale score indicates greater expression of the characteristic. The total family social capital score was calculated by summing the score for each of the family social environment scales (possible score range from 0 to 20).

### 2.3. Data Analysis

Mothers were partitioned into tertiles based on their total family social capital score. Descriptive statistics for all variables by tertiles were conducted along with determining the internal consistency of scales. ANOVA and Tukey post-hoc tests were conducted to determine how mothers’ weight-related behaviors, parenting behaviors, and health characteristics significantly differed among and between tertiles. To reduce the Type I error risk caused by multiple comparisons, the Benjamini Hochberg procedure was applied at the 5% level for 2-tailed tests, generating a probability level for ANOVA main effects of *p* ≤ 0.03 to reduce the risk of type I errors [49]. Probability for post-hoc pairwise comparisons was set at *p*< 0.05. Effect size of ANOVA main effects was determined by calculating partial eta-squared values. Effect sizes of 0.01, 0.06, and 0.14 indicated small, medium, and large effects, respectively [50]. Analytic procedures were completed with SPSS software version 27.0 (IBM Corporation, Chicago, IL, USA).

## 3. Results

Mothers in the study had a mean age of 32.65 ± 5.53 SD years and were mostly (60%) White. The majority (86%) had at least some post-secondary education. On average, family affluence was moderate (mean = 5.45 ± 1.74 SD on a 0 to 9 scale) and 36% of mothers did not have paid employment. Mothers had an average of 2 children and most (82%) lived in dual parent households.

Mothers were grouped into tertiles based on their total family social capital scores. As shown in Table 1, all tertiles differed significantly from each other on all family social capital scales with large effect sizes, thereby indicating distinct groups. As expected, scores on each scale were significantly higher in upper compared to lower tertiles indicating that family social capital was greater as tertile level incremented. Tertiles did not differ by maternal age, ethnicity/racial distribution, education level, number of children in the household, or number of parents in the household (Table 2). Mothers in Tertile 2 were significantly more likely to work full-time than Tertile 3, with a very small effect size. Tertile 1 had significantly lower family affluence than Tertile 3 (5.17 ± 1.76 SD vs. 5.71 ± 1.72 SD, an approximately 5% difference), but the effect size was small.

Data for maternal health status measures are displayed in Table 3. Higher tertile mothers tended to have significantly better health status, fewer days of “not good” physical and mental health, and lower depression severity and perceived stress than lower tertile comparators; effect sizes were small. The dietary behaviors revealed that Tertile 1 mothers tended to eat fewer fruits and vegetables and drink more sugar-sweetened beverages than other mothers. High tertile mothers tended to have significantly higher physical activity levels and, though not significant, less sedentary activity, than lower tertile mothers. Sleep quality and sleep duration were positively correlated with higher tertile assignment, with Tertile 3 scoring significantly higher than Tertile 1 mothers. However, effect sizes for dietary, physical activity, and sleep behaviors were small.

An examination of parenting behavior findings indicates that maternal modeling of healthy eating and modeling of physical activity was significantly different among and between tertiles with the highest scores in Tertile 3 and medium effect sizes (see Table 3). The child feeding practices scale scores indicated no significant differences in restriction across tertiles; however, the use of pressure was lower in higher tertile assignment, with significant differences between all pairwise comparisons and a medium effect size. Additionally, Tertile 1 was significantly more likely to use food rewards than Tertile 3, yielding a small effect size.

The home physical environment revealed that space and supports for physical activity inside the home as well as in the area outside the home was significantly higher in upper versus lower tertiles, with significant differences between all pairwise comparisons with small effect sizes (see Table 3). The home fruit/vegetable availability differed significantly among and between all pairwise comparisons with amounts greater in upper tertiles and a medium effect size. Sugar-sweetened beverage availability in households did not differ significantly among tertiles.

The child health findings displayed in Table 4 indicate that mothers’ rated children’s health as very good to excellent with the ratings being significantly greater in higher tertile assignments, with a medium effect size. Children in Tertile 3 tended to have fewer “not good” mental and physical health days than comparators. Children’s dietary intake did not differ significantly among tertiles; however, greater family social capital was associated with higher fruit/vegetable juice intake and lower sugar-sweetened beverage intake. Physical activity level was greater in higher tertiles, with Tertile 1 getting significantly less physical activity than those in Tertile 3; the effect size was small. Screen time and sleep duration did not differ significantly among tertiles. Sleep quality was significantly higher in families with greater family social capital, with Tertile 3 scoring significantly higher than other tertiles.

## 4. Discussion

This study aimed to expand the understanding of associations of family social capital with weight-related behaviors and home environments of families with young children and the potential value of incorporating strategies for building family social capital in health, nutrition, and obesity prevention interventions. The measures used to determine family social capital (i.e., supportive, engaged parenting behaviors; family cohesion; family conflict; and family meal frequency) yielded distinct tertile groups that differed significantly on every family social capital measure with large effect sizes. These differences were independent of sociodemographic characteristics. The findings indicate that greater family social capital is linked to better maternal health, dietary intake, physical activity, and sleep behaviors. In addition, maternal modeling of healthy eating and physical activity, child feeding practices, and home environments improved as family social capital increased. Child health status, mental health, physical activity, and sleep quality were also better in families with greater family social capital.

Family social capital is built through positive, supportive family relationships that teach values and societal norms [6,12]. Findings of this study suggest family social capital also confers health benefits to mothers and children which supports prior work among adolescents in Russia, Croatia, and Taiwan [51,52,53]. Mothers in families with greater social capital had better mental health, which likely offered protection to their children [54,55,56]. For instance, Reynolds and Crea reported that among U.S. families, parent depression and anxiety increased the vulnerability of adolescent children for engaging in anti-social behaviors [57]. Social capital, in the form of family cohesion, is also inversely related with disordered eating behaviors that may escalate into psychological disorders such as Anorexia Nervosa [58]. Further evidence of the effects of family social capital on children’s mental health is provided by Springer et al.’s report that close family relationships reduced the odds of teens in El Salvador from engaging in aggressive behaviors, risky behaviors such as binge drinking, drug use, and sexual relationships, and having suicidal thoughts [59].

The findings of the study reported here indicate that higher family social capital is associated with healthier maternal dietary intake, maternal role modeling of healthy eating, and household food supplies. The lack of differences in dietary intake in children contrasts with a prior report that found greater family capital in the form of family communication and role modeling was associated with better nutritional practices in Taiwan adolescents [53]. Contradictory findings may have been due to differences in measures used to assess dietary intake between studies. Similarly, a higher level of family belongingness was linked with greater fruit and vegetable intake in adolescents living in the United Kingdom [60]. The tandem increase in household availability of fruits/vegetables and family social capital supports previous work in the Philippines which found that primary food decision makers in more cohesive families had a greater propensity to make healthy food choices [6,61].

In the present study, maternal modeling of physical activity, as well as space and supports for physical activity increased as family social capital rose. In addition, physical activity level of both mothers and children tended to be higher in families with more social capital. These findings parallel reports from Taiwan where adolescents in families with better communication and adult role models had better physical activity outcomes [53]. Similarly, higher levels of family social capital were associated with regular overall physical activity in teenage Croatian boys, however this association was not observed in girls [62].

Mothers in the highest family social capital tertile group had higher quality sleep and longer sleep duration than those with the least family social capital. In fact, those in the highest family social capital group were the only mothers who met sleep recommendations of 7 to 9 h/night [63]. Children’s sleep duration did not differ among family social capital tertiles. However, children in the highest family social capital tertile had the best sleep quality, which aligns with a study of Canadian parents where greater social capital (as measured by occupation) had fewer sleep disturbances than comparators [64].

The supportiveness of the home environment for healthy eating and physical activity behaviors increased with family social capital in the study reported here. For example, mothers’ child feeding practices became more congruent with recommendations as family social capital increased. That is, restricting children’s food choices, pressuring them to eat, and using highly palatable food (e.g., sweets) to reward them for eating healthy foods (e.g., vegetables) are not recommended because they can promote or exacerbate feeding problems such as picky eating. Additionally, these feeding behaviors are not recommended because it may contribute to children’s development of an insensitivity to physiological signals of hunger and satiety that help regulate food intake amounts as well as development of preferences for highly palatable foods and reduced preference for healthy foods [65,66,67]. The home physical environment also became more supportive of health behaviors. That is, as family social capital rose so did space and supports for physical activity as well as availability of fruits/vegetables in the home. Although no comparable studies of home environments could be located, these findings are congruent with the overall premise of family social capital [6,12] in than parents in cohesive, engaged families would be inclined to create home environments supportive of optimal child development.

This study is, to the best of the authors’ knowledge, one of the first to investigate relationships of family social capital with weight-related behaviors and home environments of mothers and young children. Placing the study findings in the context of previous research is difficult for several reasons. First, despite its long history in other fields such as economics, sociology, and political science, the study of social capital in the health field began only about 20 years ago and thus there are few published studies [68]. Second, social capital is measured in a variety of ways which make direct comparisons of study results difficult. A “glaring gap in the conceptualization of social capital within the empirical literature has been the level of the family” [6]. Few studies have been conducted with children [42] or in the United States [5,6]. Additionally, many social capital studies, like the one reported here, are limited by their secondary analysis nature. That is, many published studies were not originally designed to assess social capital and, thus, are inherently constrained by available variables that are conceptually linked with social capital [36,69]. Finally, it is not possible to determine causation and/or direction of relationships due to this study being cross sectional.

Despite the study limitations, findings support the hypothesis and social capital theory [8] that family social capital is linked with positive weight-related behaviors and home environments of mothers and young children. Moreover, it suggests that strategies that teach parents how to build family social capital through amplified family verbal and physical engagement, more effective family conflict management, greater family cohesion, and more frequent family interactions such as those at mealtime could improve the effectiveness of health, nutrition, and obesity prevention interventions. Indeed, findings from the one nutrition intervention study located, which was conducted with Mexican American adults with type 2 diabetes mellitus, demonstrate it is possible to improve family social capital and, thereby, improve disease self-management [70]. Future intervention studies should consider incorporating strategies to build family social capital and compare longitudinal outcomes to traditional interventions to determine the relative value of family social capital on health behaviors [71].

## Figures and Tables

**Table 1:** Family social capital scale comparison by tertile (*n* = 557).

Family Social Capital Scales (Cronbach Alpha) #	Tertile 1 (*n* = 187) Mean ± SD (95% CI *)	Tertile 2 (*n* = 186) Mean ± SD (95% CI)	Tertile 3 (*n* = 184) Mean ± SD (95% CI)	*F* *df = 2554 ‡*	ANOVA *p*	Tukey Post-Hoc Pairwise Comparisons ^†^	Partial Eta-Squared
Verbal engagement ^A^ [44] (0.69)	1.71 ± 1.00 (1.57, 1.86)	2.19 ± 0.83 (2.07, 2.31)	2.73 ± 0.90 (2.60, 2.86)	57.181	<0.001	ABC	0.171
Physical engagement ^A^ [44] (n/a)	3.07 ± 1.03 (2.93, 3.22)	3.62 ± 0.57 (3.54, 3.70)	3.85 ± 0.42 (3.79, 3.91)	56.233	<0.001	ABC	0.169
Family conflict ^A^ [45,46,47] (0.86)	2.27 ± 0.98 (2.13, 2.41)	2.95 ± 0.85 (2.83, 3.07)	3.62 ± 0.66 (3.53, 3.72)	120.262	<0.001	ABC	0.303
Family cohesion ^A^ [45,46,47] (0.87)	2.54 ± 0.78 (2.43, 2.65)	3.12 ± 0.52 (3.05, 3.20)	3.63 ± 0.45 (3.57, 3.70)	153.128	<0.001	ABC	0.356
Family meal frequency ^B^ (n/a)	1.80 ± 1.02 (1.65, 1.95)	2.52 ± 0.97 (2.38, 2.66)	3.12 ± 0.83 (3.00, 3.24)	90.294	<0.001	ABC	0.246

# Cronbach alpha. * CI = Confidence Interval *‡ df = Degrees of Freedom* ^†^ Pairwise comparisons A = Tertile 1 and 2 differed significantly; B = Tertile 1 and 3 differed significantly; C = Tertile 2 and 3 differed significantly (*p* < 0.05). ^A^ 5-point agreement rating: Strongly disagree, disagree, neither agree nor disagree, agree, strongly agree; scored 0 to 4, respectively, with scoring reversed for negatively worded statements; scale score equals average of item scores; higher scale score indicates greater expression of the characteristic. ^B^ Days/week of having family meals at breakfast, lunch, and dinner. Total possible range = 0 to 4; scored as 0 if <5 meals/week, 1 if ≥5 to <9 meals/week, 2 if ≥9 to <13, 3 if ≥13 to <17 meals/week, 4 if ≥17 meals/week.

**Table 2:** Maternal sociodemographic characteristic comparisons by family social capital tertiles (*n* = 557).

Family Social Capital Scales	Tertile 1 (*n* = 187) Mean ± SD (95% CI *)	Tertile 2 (*n* = 186) Mean ± SD (95% CI)	Tertile 3 (*n* = 184) Mean ± SD (95% CI)	*F* *df = 2554* ‡	ANOVA *p*	Tukey Post-Hoc Pairwise Comparisons ^†^	Partial Eta-Squared
Age	32.51 ± 5.78 (31.68, 33.34)	32.82 ± 5.42 (32.03, 33.60)	32.61 ± 5.43 (31.82, 33.40)	0.147	0.864		0.001
Race/ethnicity (white vs. non-white) ^A^	0.42 ± 0.50 (0.35, 0.49)	0.38 ± 0.49 (0.31, 0.45)	0.39 ± 0.49 (0.31, 0.46)	0.461	0.631		0.002
Highest level of education ^B^	2.30 ± 0.71 (2.20, 2.41)	2.41 ± 0.69 (2.31, 2.51)	2.33 ± 0.73 (2.22, 2.44)	1.065	0.345		0.004
Number of children Under 18 in the household	2.35 ± 1.16 (2.18, 2.51)	2.23 ± 1.11 (2.07, 2.39)	2.16 ± 1.06 (2.00, 2.31)	1.399	0.248		0.005
Marital status (single or dual parent household) ^C^	1.78 ± 0.41 (1.72, 1.84)	1.86 ± 0.35 (1.81, 1.91)	1.81 ± 0.39 (1.75, 1.87)	2.019	0.134		0.007
Maternal employment ^D^	1.17 ± 0.92 (1.03, 1.30)	1.21 ± 0.86 (1.09, 1.33)	0.97 ± 0.93 (0.83, 1.10)	3.770	0.024	C	0.013
Family affluence scale ^E^	5.17 ± 1.76 (4.92, 5.43)	5.49 ± 1.71 (5.24, 5.74)	5.71 ± 1.72 (5.46, 5.96)	4.501	0.012	B	0.016

* CI = Confidence Interval ‡ *df =* Degrees of Freedom ^†^ Pairwise comparisons A = Tertile 1 and 2 differed significantly; B = Tertile 1 and 3 differed significantly; C = Tertile 2 and 3 differed significantly (*p* < 0.05). ^A^ Coded 0 = white; 1 = non-white; ^B^ Coded 1 = high school or less; 2 = some post-secondary education; 3 = college degree or higher; ^C^ Coded 1 = single parent; 2 = dual parent; ^D^ Coded 0 = no paid employment; 1 = part-time paid employment; 2 = full-time paid employment; ^E^ scale scored 0 to 9 points; greater points indicate greater family affluence.

**Table 3:** Maternal health, weight-related behaviors, and home environment comparisons by family social capital tertiles (*n* = 557).

Measures	Tertile 1 (*n* = 187) Mean ± SD (95% CI *)	Tertile 2 (*n* = 186) Mean ± SD (95% CI)	Tertile 3 (*n* = 184) Mean ± SD (95% CI)	*F* *df = 2554* ‡	ANOVA *p*	Tukey Post-Hoc Pairwise Comparisons ^†^	Partial Eta-Squared
**Health**							
Health status ^A^ [26,27]	3.28 ± 0.95 (3.15, 3.42)	3.37 ± 0.86 (3.25, 3.42)	3.72 ± 1.02 (3.58, 3.87)	11.216	<0.001	BC	0.039
Physical health quality of life ^B^ [26,27]	3.87 ± 6.79 (2.89, 4.85)	3.20 ± 5.07 (2.47, 3.93)	2.29 ± 5.23 (1.53, 3.05)	3.538	0.030	B	0.013
Mental health quality of life ^B^ [26,27]	6.70 ± 8.64 (5.45, 7.95)	4.83 ± 7.13 (3.80, 5.86)	3.46 ± 6.34 (2.54, 4.38)	8.871	<0.001	AB	0.031
Depression severity ^C^ [28]	1.74 ± 0.77 (1.63, 1.85)	1.57 ± 0.71 (1.47, 1.67)	1.52 ± 0.73 (1.41, 1.62)	4.541	0.011	B	0.016
Perceived stress ^C^ [29]	1.76 ± 0.79 (1.64, 1.87)	1.59 ± 0.78 (1.48, 1.70)	1.43 ± 0.67 (1.33, 1.53)	8.970	<0.001	AB	0.042
**Dietary Intake**							
Fruit/vegetable (serv/day) [30]	4.01 ± 1.93 (3.73, 4.29)	4.47 ± 1.68 (4.22, 4.71)	5.05 ± 2.00 (4.60, 5.17)	10.377	<0.001	AB	0.036
Sugar-sweetened beverages (serv/day) [25]	0.85 ± 0.91 (0.72, 0.98)	0.74 ± 0.81 (0.62, 0.86)	0.61 ± 0.77 (0.49, 0.72)	4.028	0.018	B	0.014
**Physical Activity** [31]							
Physical activity level ^D^ [25,31]	12.15 ± 9.21 (10.82, 13.48)	13.98 ± 10.07 (12.52, 15.43)	16.59 ± 9.66 (15.18, 17.99)	9.891	<0.001	BC	0.034
Screen time (min/day)	383.82 ± 289.32 (342.08, 425.56)	351.45 ± 287.70 (309.83, 393.07)	315.57 ± 248.85 (279.38, 351.77)	2.837	0.059		0.010
**Sleep** [32,33]							
Sleep quality ^E^	3.04 ± 0.88 (2.92, 3.17)	3.19 ± 0.95 (3.05, 3.33)	3.32 ± 0.87 (3.19, 3.45)	4.391	0.013	B	0.016
Sleep duration (hours/day)	6.93 ± 1.27 (6.75, 7.12)	6.99 ± 1.28 (6.81, 7.18)	7.28 ± 1.16 (7.11, 7.18)	4.027	0.018	B	0.015
**Parenting Behaviors** [23,24,25]							
Modeling of healthy eating ^F^	3.33 ± 0.82 (3.22, 3.45)	3.62 ± 0.71 (3.52, 3.73)	3.93 ± 0.76 (3.82, 4.04)	28.232	<0.001	ABC	0.091
Modeling of physical activity (days/week)	2.73 ± 1.22 (2.56, 2.91)	3.26 ± 1.19 (3.09, 3.43)	3.59 ± 1.29 (3.40, 3.78)	22.435	<0.001	ABC	0.075
**Child Feeding Practices ^F^**							
Parent feeding: restriction	3.77 ± 0.82 (3.65, 3.89)	3.77 ± 0.82 (3.65, 3.89)	3.86 ± 1.00 (3.72, 4.01)	0.724	0.485		0.003
Parent feeding: pressure	2.58 ± 0.95 (2.44, 2.71)	2.27 ± 0.90 (2.14, 2.40)	2.01 ± 0.96 (1.87, 2.15)	17.232	<0.001	ABC	0.059
Parent feeding: instrumental (food reward)	2.50 ± 0.72 (2.39, 2.60)	2.38 ± 0.72 (2.27, 2.48)	2.28 ± 0.80 (2.16, 2.40)	3.903	0.021	B	0.014
**Home Physical Activity Environment** [34]							
Indoor home physical activity space and supports ^F,G^	3.14 ± 0.84 (3.02, 3.26)	3.35 ± 0.84 (3.22, 3.47)	3.56 ± 0.77 (3.45, 3.67)	12.457	<0.001	ABC	0.043
Outdoor/yard physical activity space and supports ^G^	4.22 ± 0.72 (4.11, 4.34)	4.39 ± 0.64 (4.30, 4.49)	4.56 ± 0.56 (4.47, 4.64)	11.276	<0.001	ABC	0.043
**Home Food Environment [35]**							
Fruit/vegetables (serv available/household member/week)	5.41 ± 1.99 (5.12, 5.69)	5.97 ± 1.86 (5.70, 6.24)	6.58 ± 2.14 (6.27, 6.89)	16.051	<0.001	ABC	0.055
Sugar-sweetened beverages (serv available /household member/week)	1.79 ± 1.84 (1.52, 2.05)	1.74 ± 1.82 (1.48, 2.01)	1.49 ± 1.82 (1.22, 1.75)	1.465	0.232		0.005

* CI = Confidence Interval ‡ *df =* Degrees of Freedom ^†^ Pairwise comparisons A = Tertile 1 and 2 differed significantly; B = Tertile 1 and 3 differed significantly; C = Tertile 2 and 3 differed significantly. ^A^ 5-point excellence rating: poor, fair, good, very good, excellent; scored 1 to 5, respectively. ^B^ Days/month of “not good” health ^C^ 4-point frequency rating: not at all, several days, more than half the days, nearly every day; scored 1 to 4. Items averaged to create scale score. Cronbach alpha for Depression Severity and Perceived Stress are 0.74 and 0.78, respectively. ^D^ Days/week engaging in walking, moderate, or vigorous activity, for at least 10 min; days weighted by intensity of 1, 2, and 3, respectively, and summed; score range 0 to 42. ^E^ 5-point rating scale: very bad, bad, okay, good, very good; scored 1 to 5, respectively. ^F^ 5-point agreement rating: strongly disagree, disagree, neither agree nor disagree, agree, strongly agree; scored 1 to 5, respectively; scale score equals average of item scores. Cronbach alpha for modeling of healthy eating, parent feeding: pressure, parent feeding: instrumental, indoor home physical activity space and supports, and outdoor/yard physical activity space and supports are 0.74, 0.69, 0.74, 0.71, and 0.74, respectively. Cronbach alpha for other scales sharing this superscript could not be calculated due to scale length (<2 items) and/or scale type. ^G^ 5-point occurrence rating: almost never, 1–2 times/week; 3 to 4 times/week, 5 to 6 times/week, every day; scored 1 to 5, respectively; scale score equals average of item scores.

**Table 4:** Child health and weight-related behaviors comparisons by family social capital tertiles (*n* = 557).

Measures	Tertile 1 (*n* = 187) Mean ± SD (95% CI *)	Tertile 2 (*n* = 186) Mean ± SD (95% CI)	Tertile 3 (*n* = 184) Mean ± SD (95% CI)	*F* *df = 2554* ‡	ANOVA *p*	Tukey Post-Hoc Pairwise Comparisons ^†^	Partial Eta-Squared
**Child Health Status ^A^** [26,27]	4.16 ± 0.77 (4.04, 4.27)	4.37 ± 0.86 (4.25, 4.49)	4.64 ± 0.64 (4.55, 4.73)	19.034	<0.001	ABC	0.055
Physical health quality of life ^B^	1.33 ± 2.57 (0.96, 1.70)	1.96 ± 4.77 (1.27, 2.65)	1.17 ± 2.85 (1.19, 1.78)	2.559	0.078		0.009
Mental health quality of life ^B^	2.66 ± 4.47 (2.01, 3.30)	2.83 ± 5.43 (2.04, 3.61)	1.68 ± 1.97 (1.40, 1.97)	3.941	0.020	C	0.014
**Child Dietary Intake**							
Fruit/vegetable juice (serv/day) [30]	0.68 ± 0.50 (0.61, 0.75)	0.65 ± 0.50 (0.57, 0.72)	0.75 ± 0.56 (0.67, 0.83)	1.777	0.170		0.007
Sugar-sweetened beverages (serv/day) [25]	0.38 ± 0.50 (0.31, 0.45)	0.33 ± 0.46 (0.26, 0.39)	0.27 ± 0.41 (0.21, 0.33)	2.776	0.063		0.006
**Physical Activity** [31]							
Physical activity level ^C^	23.82 ± 12.35 (22.04, 25.60)	26.56 ± 10.91 (24.99, 28.14)	28.2 ± 10.82 (26.63, 29.77)	7.027	0.001	B	0.021
Screen time (min/day)	317.57 ± 293.38 (275.24, 359.89)	292.82 ± 260.59 (255.13, 330.52)	274.89 ± 261.55 (236.85, 312.93)	1.149	0.318		0.003
**Child Sleep ^D^** [32,33]							
Sleep quality	4.09 ± 0.78 (3.97, 4.20)	4.18 ± 0.80 (4.07, 4.30)	4.48 ± 0.652 (4.38, 4.57)	7.740	<0.001	BC	0.033
Sleep duration (hours/day)	10.31 ± 1.34 (10.10, 10.52)	10.60 ± 1.50 (10.37, 10.83)	10.60 ± 1.38 (10.39, 10.81)	2.302	0.101		0.009

* CI = Confidence Interval ‡ *df =* Degrees of Freedom ^†^ Pairwise comparisons A = Tertile 1 and 2 differed significantly; B = Tertile 1 and 3 differed significantly; C = Tertile 2 and 3 differed significantly ^A^ 5-point excellence rating: poor, fair, good, very good, excellent; scored 1 to 5, respectively. ^B^ Days/month of “not good” health. ^C^ Days/week engaging in walking, moderate, or vigorous activity, for at least 10 min; days weighted by intensity of 1, 2, and 3, respectively, and summed; score range 0 to 42. ^D^ 5-point rating scale: very bad, bad, okay, good, very good; scored 1 to 5, respectively.

## Data Availability

Data sharing not applicable. No new data were created or analyzed in this study. Data sharing is not applicable to this article.
